# Lipoproteins and cholesterol homeostasis in paediatric nephrotic syndrome patients

**DOI:** 10.11613/BM.2022.020706

**Published:** 2022-06-15

**Authors:** Yonas Mulat Simachew, Tamara Antonić, Tamara Gojković, Sandra Vladimirov, Marija Mihajlović, Sanja Vujčić, Gordana Miloševski-Lomić, Jelena Vekić, Aleksandra Zeljković, Vesna Spasojević-Kalimanovska, Amira Peco-Antić, Dušan Paripović, Aleksandra Stefanović

**Affiliations:** 1 Department of Medical Biochemistry, Faculty of Pharmacy, University of Belgrade, Belgrade, Serbia; 2 Department of Nephrology, University Children’s Hospital, Belgrade, Serbia; 3 School of Medicine, University of Belgrade, Belgrade, Serbia

**Keywords:** paediatric nephrotic syndrome, glucocorticoid therapy, serum lipid profile, non-cholesterol sterols

## Abstract

**Introduction:**

The aim of this study was to investigate lipoprotein particle distributions and the likelihood of achieving cholesterol homeostasis in the remission phase of nephrotic syndrome (NS) in paediatric patients. We hypothesized that lipoprotein particle distributions moved toward less atherogenic profile and that cholesterol homeostasis was achieved.

**Materials and methods:**

Thirty-three children, 2 to 9 years old with NS were recruited. Blood sampling took place both in the acute phase and during remission. Serum low-density lipoprotein particles (LDL) and high-density lipoprotein particles (HDL) were separated using non-denaturing polyacrylamide gradient gel (3-31%) electrophoresis. Serum non-cholesterols sterols (NCSs), desmosterol, lathosterol, 7-dehydrocholesterol (7-DHC), campesterol and β-sitosterol were measured by high-performance liquid chromatography-tandem mass spectrometry (HPLC-MS/MS).

**Results:**

All patients had desirable serum HDL cholesterol concentrations during remission. The dominant lipoprotein diameters and LDL subclass distribution did not change significantly during follow-up. In contrast, HDL lipoprotein particle distribution shifted towards larger particles. The absolute concentration of desmosterol was significantly lower during remission (P = 0.023). β-sitosterol concentration markedly increased during remission (P = 0.005). Desmosterol/β-sitosterol (P < 0.001) and 7-DHC/β-sitosterol (P = 0.005) ratios significantly declined during disease remission.

**Conclusions:**

Favourable changes in the serum lipid profiles, HDL particle subclass distribution and cholesterol metabolism in paediatric patients with NS during remission took place. For the first time, we found that cholesterol homeostasis changed in favour of increased cholesterol absorption during disease remission. Nevertheless, complete cholesterol homeostasis was not achieved during disease remission.

## Introduction

Nephrotic syndrome (NS) is a glomerular disorder affecting between 2-7 *per* 100,000 children below 16 years of age worldwide (*1*). It is an idiopathic condition in paediatric patients characterized by proteinuria, hypoalbuminemia, oedema and hyperlipidaemia (*1*). In response to glucocorticoid therapy, clinically idiopathic NS is defined as steroid-sensitive or steroid-resistant. The majority of paediatric idiopathic NS is steroid-sensitive which means that the symptoms of proteinuria, hypoalbuminemia and oedema will decrease within a few weeks of therapy (remission). However, approximately 50% of the patients will develop frequent disease relapses (*1*).

Nephrotic syndrome results in increased concentrations of total serum cholesterol (TC), low-density lipoprotein cholesterol (LDL-C), triglycerides (TG) and a variable concentration of serum high-density lipoprotein cholesterol (HDL-C) (*2*). These lipid disturbances in nephrotic patients are usually resolved with proteinuria therapy (high dose corticosteroid treatment) but a significant number of patients have persistent hyperlipidaemia even during disease remission (*3*-*7*). Also, NS significantly affects the composition and function of LDL and HDL particles, and may increase their atherogenic potential (*2*). Small HDL3 subclasses - HDL3b, HDL3a and HDL3c are more active in promoting cholesterol efflux, have greater anti-inflammatory and anti-oxidant properties in the healthy population (*8*). However, in atherosclerotic related diseases with hypertriglyceridemia they lose their protective properties (*8*). Few studies have shown the presence of more abundant HDL3 particles in the active phase of NS, whereas studies during remission phase are not available (*9*, *10*). Likewise, several LDL subclasses have been identified and smaller subclasses LDL IIIA, LDL IIIB, LDL IVA and LDL IVB are considered as atherogenic (small, dense LDL (sdLDL)) (*11*). The sdLDL particles are atherogenic presumably due to their relatively higher susceptibility to oxidation and higher penetrative capacity of the endothelial barrier (*11*). There are no existing data about the prevalence of sdLDL particles in paediatric NS patients.

Although an increased serum LDL-C concentration is the prominent feature of NS, its origin is not fully understood. Studies suggest that increased synthesis and decreased LDL particle catabolism contribute to hypercholesterolemia in NS patients (*2*). Serum non-cholesterol sterols (NCSs) measurement can reveal whether hypercholesterolemia is due in part to increased hepatic synthesis, increased intestinal absorption, combination of both, or neither (*12*). Among the most common NCSs, desmosterol, lathosterol and 7-dehydrocholesterol (7-DHC) are used as markers of cholesterol synthesis, whereas β-sitosterol and campesterol are used as markers of intestinal cholesterol absorption (*13*-*15*). Recently published results indicate that in children aged 1 to 10 years without dyslipidaemia, cholesterol absorption is the dominant process in the cholesterol metabolic pathway (*14*). To date, information is not available on cholesterol homeostasis in paediatric nephrotic patients.

This study aimed to evaluate serum lipid concentrations, lipoprotein particle distribution, cholesterol synthesis and absorption markers in the acute and the remission phase of paediatric NS. Our primary hypothesis was that lipoprotein particle distributions moved toward less atherogenic profile and that cholesterol homeostasis was achieved in remission phase of disease.

## Materials and methods

### Subjects

A cohort of paediatric patients with idiopathic steroid-sensitive NS comprising of 22 boys and 11 girls was recruited prospectively from 2016 to 2019. The median age of the patients was 5 years (range, 2–9 years). All patients were recruited from the University Children’s Hospital in Belgrade – “Tirsova”. The diagnosis of NS, corticosteroid treatment and definition of the remission phase were carried out according to the protocols of the Kidney Disease Improving Global Outcomes (KDIGO) guidelines (*16*). None of the patients was treated at the time of blood sampling for the first point of study investigation (acute-phase point). After high dose prednisolone treatment in the acute phase (2 mg/kg/day for 4 weeks (maximum 60 mg) - to induce remission), patients were treated with low dose alternate-day prednisolone treatment during remission. All patients achieved remission within a median of 12 (9-15) days. At the time of the second sample collection the average dose of prednisolone treatment for patients was 1.5 mg/kg/day (40 mg/m^2^/day). None of the patients received lipid-lowering therapy. Body mass index (BMI) calculator computed the BMI for children. Seated blood pressure measurement was taken by auscultatory method using mercury sphygmomanometer with an appropriate cuff size. The children’s caregivers gave informed consent and the study was carried out under guidelines described in the Helsinki Declaration. The ethics committee of the University of Belgrade Children’s Hospital – “Tirsova” approved the study protocol.

### Methods

#### Blood collection and basic biochemical analyses

Blood sampling for this study was performed in the acute phase (before therapy started) and during remission. The average time between the first and the second sampling point was 40 (IQR 30-50) days. Blood samples were collected into serum sample vacutainer tubes after a 12-hour fasting period. The serum was obtained by immediate centrifugation at 1500xg for 10 minutes at 4 °C. Aliquots were stored at – 80 °C and thawed immediately before analyses. Glucose, urea, creatinine, total protein, albumin, TC, TG and HDL-C concentrations were quantified by routine methods on an ILab 300+ analyser (Instrumentation Laboratory, Milan, Italy). Serum LDL-C concentrations were estimated using Friedewald’s formula (LDL-C = TC - HDL - C - TG/2.2). The target lipid concentrations during remission were assessed based on the National Heart, Lung and Blood Institute (NHLBI) guidelines, which recommend screening for hyperlipidaemia in children with diabetes mellitus and other conditions predisposing to the development of accelerated atherosclerosis (including NS) (*17*).

#### Determination of LDL and HDL subclasses

Separation of serum LDL and HDL particles was performed by non-denaturing polyacrylamide gradient gel (3-31%) electrophoresis. After electrophoretic separation, the gel was scanned to examine lipoprotein subclasses using the Image Scanner (Amersham Pharmacia Biotech, Vienna, Austria) with Image Quant software (version 5.2; 1999; Molecular Dynamics, Sunnyvale, USA). The dominant LDL and HDL particle diameter was defined by estimating the diameter of the major peak in their regions in the densitogram. The relative proportions of each LDL and HDL subclasses were estimated by determining the areas under the peaks after densitometric scans of the samples. The relative proportion of sdLDL was estimated by computing the area of the densitometric scan at or below 25.5 nm. Procedure for determining LDL and HDL subclasses was done according to Rainwater *et al.* (*18*).

#### Determination of the serum concentration of non-cholesterol sterols

Non-cholesterol sterols were quantified using high-performance liquid chromatography-tandem mass spectrometry (HPLC-MS/MS). A detailed description of the procedure has been published elsewhere (*19*). We analysed the following non-cholesterol sterols: desmosterol, lathosterol, 7-DHC, β-sitosterol and campesterol. Serum concentrations of non-cholesterol sterols were expressed in μmol/L (absolute concentration) and their ratio to cholesterol (mmol/mol of cholesterol) by adjusting the non-cholesterol sterol concentrations to the total cholesterol value (relative concentrations). The use of the sterol serum concentration to cholesterol ratio as a marker of cholesterol synthesis and absorption has been recommended to eliminate the effect of variations in the serum cholesterol concentrations on the non-cholesterol concentration (*20*). This study used serum desmosterol/TC, lathosterol/TC and 7-DHC/TC ratio as the cholesterol synthesis marker, whereas serum β-sitosterol/TC and campesterol/TC ratios as cholesterol absorption markers (*13*-*15*). We also calculated the synthesis/absorption marker ratios, which reflect overall cholesterol metabolism (*14*, *15*).

### Statistical analysis

Due to small sample size (N = 33), data are presented as median (IQR, interquartile range), while differences in parameters between acute and remission phase of NS, were evaluated by using the Wilcoxon signed ranks test. Correlation analysis was performed by Spearman’s correlation test. All data were analysed using SPSS Statistics version 22 software. A P-value of less than 0.05 was considered statistically significant.

## Results

Anthropometric and basic biochemical parameters are shown in Table 1. The median age of the patients was 5 years (range; 2–9 years), and the male-to-female ratio was 22:11. There were no significant differences in systolic blood pressure (SBP), diastolic blood pressure (DBP), glucose, urea or creatinine between the acute and NS remission phase. As expected, serum total protein and albumin concentrations were significantly higher in remission (P = 0.001 and P < 0.001). In remission phase, a significant decrease of TC, LDL-C, TG, and increase of HDL-C concentrations were observed (P < 0.001 for TC, LDL-C and HDL-C; and for TG P = 0.013). After a case by case analysis, according to the recommendations of NHLBI guidelines, we found the following results: 0.91 of patients had a high (≥ 5.2 mmol/L), 0.06 had a borderline (4.4 - 5.2 mmol/L) and 0.03 had an acceptable (< 4.4 mmol/L) TC concentration in remission (*17*). More than half of the patients (0.56) had a high (≥ 3.4 mmol/L), 0.22 had a borderline (2.8-3.3 mmol/L) and 0.22 had an acceptable (< 2.8 mmol/L) LDL-C concentration while 0.94 of children had an acceptable (> 1.2 mmol/L), 0.06 had a borderline (1.0 - 1.2 mmol/L) and none had an abnormal (< 1.0 mmol/L) HDL-C concentration. A proportion of 0.64 children had a high (≥ 1.1 mmol/L), 0.18 had a borderline (0.8-1.1 mmol/L) and 0.18 had acceptable (< 0.8 mmol/L) TG concentration.

**Table 1:** Anthropometric and basic biochemical parameters in children with nephrotic syndrome in acute phase and remission

**Parameters**	**NS - acute phase** **(N = 33)**	**NS - remission** **(N = 33)**	**P**
Male (N, proportion)	22 (0.67)	22 (0.67)	1.000
BMI, kg/m^2^	18 (17-19)	17 (15-18)	0.001
SBP, mmHg	110 (99-120)	103 (100-119)	0.635
DBP, mmHg	70 (60-80)	68 (60-79)	0.422
Glucose, mmol/L	5.1 (4.3-6.1)	4.8 (4.3-5.3)	0.069
Urea, mmol/L	4.3 (3.3-5.9)	4.5 (4.1-5.7)	0.820
Creatinine, μmol/L	34 (26-41)	39 (31-49)	0.086
Total protein, g/L	42 (38-48)	62 (57-68)	0.001
Albumin, g/L	11 (8-18)	31 (26-36)	< 0.001
TC, mmol/L	9.5 (7.1-11.6)	7.1 (6.0-8.3)	< 0.001
Acceptable < 4.4, (N, proportion)	0 (0)	1 (0.03)	
Borderline-High 4.4-5.2, (N, proportion)	0 (0)	2 (0.06)	
High ≥ 5.2, (N, proportion)	33 (1.00)	30 (0.91)	
LDL-C, mmol/L	7.1 (4.6 - 8.9)	3.6 (2.8 - 5.5)	< 0.001
Acceptable < 2.8, (N, proportion)	0 (0)	7 (0.22)	
Borderline-high 2.8-3.3. (N, proportion)	3 (0.09)	7 (0.22)	
High ≥ 3.4, (N, proportion)	30 (0.91)	19 (0.56)	
HDL-C, mmol/L	1.3 (0.9-1.7)	2.2 (1.8-2.8)	< 0.001
Acceptable > 1.2, (N, proportion)	18 (0.55)	31 (0.94)	
Borderline-high 1.0-1.2, (N, proportion)	5 (0.15)	2 (0.06)	
Low < 1.0, (N, proportion)	10 (0.30)	0 (0)	
TG, mmol/L	2.0 (1.3-2.9)	1.7 (1.0-2.0)	0.013
Acceptable < 0.8, (N, proportion)	4 (0.12)	6 (0.18)	
Borderline-high 0.8-1.1, (N, proportion)	4 (0.12)	6 (0.18)	
High ≥ 1.1, (N, proportion)	25 (0.76)	21 (0.64)	
Data are expressed as number (proportion) and median (interquartile range). Data were compared using the Wilcoxon signed rank test. BMI - body mass index. SBP - systolic blood pressure. DBP - diastolic blood pressure. LDL-C - low density lipoprotein cholesterol. HDL-C - high density lipoprotein cholesterol. TC - total cholesterol. TG – triglycerides. P < 0.05 was considered statistically significant.			

Table 2 shows LDL and HDL particle size and subclass distribution in acute and remission phases. There were no significant alterations in the dominant diameters and subclass distribution of LDL particles during the course of the disease (Table 2). In contrast, increased proportions of large-size HDL particles (HDL2b and 2a) (P = 0.013 and P = 0.005, respectively) and a reduced proportion of small-sized HDL particles (HDL3c) were observed during remission compared with the acute phase (P = 0.001). There was no significant difference in dominant HDL particle diameter between the acute and remission phases (Table 2).

**Table 2:** LDL and HDL particles size and subclass distribution in children with nephrotic syndrome in acute phase and remission

**Parameters**	**NS – acute phase** **(N = 33)**	**NS – remission** **(N = 33)**	**P**
LDL diameter, nm	26.60 (25.60-26.97)	26.32 (25.60-26.94)	0.810
LDL I, %	21 (16-26)	21 (18-24)	0.631
LDL IIA,%	13 (11-16)	14 (12-16)	0.923
LDL IIB,%	15 (13-19)	17 (14-19)	0.239
LDL II,%	30 (26-34)	31 (25-34)	0.414
LDL IIIA,%	13 (11-16)	14 (12-16)	0.230
LDL IIIB,%	7 (6-9)	7 (6-8)	0.239
LDL III,%	19 (18-23)	22 (18-24)	0.130
LDL IVA, %	11 (10-15)	11 (10-13)	0.810
LDL IVB,%	15 (11-19)	14 (12-18)	0.648
LDL IV,%	26 (21-32)	27 (23-30)	0.904
sdLDL,%	46 (41-54)	47 (44-52)	0.943
HDL diameter, nm	8.59 (8.09-10.59)	9.08 (8.50-10.15)	0.325
HDL 2b,%	33 (28-39)	38 (29-43)	0.013
HDL 2a,%	20 (17-21)	21 (20-23)	0.005
HDL 3a,%	17 (14-19)	17 (15-19)	0.517
HDL 3b,%	12 (9-15)	12 (9-14)	0.149
HDL 3c,%	17 (14-22)	13 (10-16)	0.001
Data are expressed as median (interquartile range) and were compared using the Wilcoxon signed rank test. LDL - low density lipoprotein. HDL-C - high density lipoprotein. P < 0.05 was considered statistically significant.			

**Figure 1:**
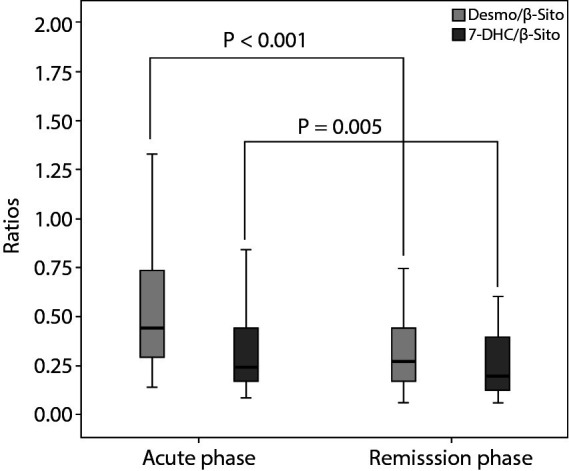
Cholesterol synthesis-absorption ratios in paediatric patients with steroid sensitive nephrotic syndrome. Desmo/β-sito - desmosterol to β-sitosterol. 7-DHC/β-sito - 7-dehydrocholesterol to β-sitosterol. Data are presented as medians with interquartile range. Data were compared by using Wilcoxon signed ranks test. P < 0.05 was considered statistically significant

In Table 3 serum cholesterol synthesis and absorption markers are shown. Their values are expressed both in absolute concentrations and as a ratio to total cholesterol (relative concentration). Among the cholesterol synthesis markers, the absolute concentration of desmosterol was significantly lower in the disease remission phase than in the acute phase (P = 0.023). However, the difference was lost when desmosterol was standardized to TC. Although the relative concentration of 7-DHC was significantly higher in remission than in the acute phase (P = 0.043), its absolute concentration was not significantly different (P = 0.710). Regarding absorption markers, only β-sitosterol (both absolute and relative concentration) was markedly higher in remission compared with the acute phase (P = 0.005 for the absolute value and P < 0.001 for the relative value). To assess cholesterol homeostasis, we calculated the ratio of synthesis and absorption markers. There was a significant decrease in desmosterol/β-sitosterol and 7-DHC/β-sitosterol ratios in patients in remission compared with the acute phase (P < 0.001 and P = 0.005, respectively) (Figure 1). Other NCS ratios did not show significant changes (data not shown). Finally, relationships within the non-cholesterol sterols were evaluated to determine whether cholesterol homeostasis was intact. We found an inverse association between cholesterol synthesis and absorption markers, both during acute and remission phases. However, the correlation coefficients were not found to be statistically significant (Table 4).

**Table 3:** Non-cholesterol sterols in children with nephrotic syndrome in acute phase and remission

**Parameters**	**NS - acute phase** **(N = 33)**	**NS – remission** **(N = 33)**	**P**
**Cholesterol synthesis markers (absolute concentration)**			
Desmosterol, μmol/L	3.88 (2.67-5.12)	2.90 (2.13-4.12)	0.023
7-dehydroholesterol, μmol/L	2.15 (1.61-2.66)	2.21 (1.89-2.91)	0.710
Lathosterol, μmol/L	5.98 (3.59-8.18)	5.34 (4.00-8.29)	0.860
**Cholesterol absorption markers (absolute concentration)**			
Campesterol, μmol/L	4.13 (2.56-5.38)	4.80 (2.74-6.31)	0.926
β-sitosterol, μmol/L	8.74 (5.32-11.36)	11.05 (7.66-16.57)	0.005
**Cholesterol synthesis markers (relative concentration)**			
Desmosterol/TC, mmol/mol	0.46 (0.32-0.56)	0.45 (0.30-0.61)	0.940
7-dehydrocholesterol/TC, mmol/mol	0.24 (0.18-0.38)	0.32 (0.23-0.49)	0.043
Lathosterol/TC, mmol/mol	0.67 (0.38-1.02)	0.81 (0.54-1.46)	0.165
**Cholesterol absorption markers (relative concentration)**			
Campesterol/TC, mmol/mol	0.48 (0.31-0.59)	0.69 (0.38-0.98)	0.082
β-sitosterol/TC, mmol/mol	0.90 (0.65-1.05)	1.31 (1.03-2.36)	< 0.001
Data are expressed as median (Interquartile range) and were compared by using Wilcoxon signed rank test. TC - total cholesterol. NS - nephrotic syndrome. P < 0.05 was considered statistically significant.			

**Table 4:** Correlation between cholesterol homeostasis markers in acute phase and remission of paediatric nephrotic syndrome

	**Acute phase NS**		**Remission phase NS**	
**Parameters**	**r_s_**	**P**	**r_s_**	**P**
Desmosterol-Campesterol	- 0.25	0.176	- 0.07	0.699
Desmosterol-β sitosterol	- 0.11	0.564	+ 0.10	0.583
7DHC-Campesterol	- 0.31	0.090	- 0.18	0.328
7DHC-β sitosterol	- 0.20	0.282	- 0.04	0.827
Lathosterol-Campesterol	- 0.21	0.254	- 0.14	0.436
Lathosterol-β sitosterol	- 0.29	0.101	+ 0.03	0.872
r_s_ – Spearman correlation coefficient. 7-DHC - 7-dehydrocholesterol. NS - nephrotic syndrome.				

## Discussion

In this prospective study, for the first time we conducted in-depth analysis of lipid and lipoproteins in paediatric NS patients. The main findings were as follows: a) in remission phase, 0.91 of patients had high TC, 0.56 of patients had high LDL-C and 0.64 had high TG concentrations, while all patients had desirable HDL-C concentration; b) the size and subclasses distribution of LDL particles in remission phase were similar to the acute phase, whereas a shift towards large-sized HDL subclass distribution was observed during remission; c) markers of cholesterol synthesis (7-DHC/TC) and markers of cholesterol absorption (β-sitosterol/TC) were increased during remission with cholesterol absorption predominates over cholesterol synthesis during remission ; d) cholesterol homeostasis was not achieved even during remission phase.

Although serum lipid profile was significantly improved during remission, significant number of patients still had hyperlipidemia. This persisting lipid abnormalities are in agreement with results of previous studies (*3*, *4*). Patients were on low-dose corticosteroid therapy. Other studies also reported persistent lipid abnormalities during a prolonged period of disease remission (*5*-*7*). According to the results from previous studies, hyperlipidaemia during remission predicts relapse in idiopathic NS (*21*, *22*). The survival rate of children with NS has remarkably improved with corticosteroid therapy. However, the occurrence of hyperlipidaemia and its associated morbidity are of great concern (*7*).

LDL and HDL particles are vastly heterogeneous in terms of their physicochemical properties and functions. Small HDL subclasses are generally more active in promoting cholesterol efflux and have both greater anti-inflammatory and anti-oxidant properties in the healthy population (*8*). However, increased small HDL in serum in different diseases may be due to irregularity in the maturation of HDL and impairment of reverse cholesterol transport, which may increase the risk of atherosclerotic cardiovascular disease (*8*). Several studies have demonstrated that large, α-migrating HDL is the best negative predictor of recurrent cardiovascular events, while smaller α-migrating HDL positively predicts such events (*23*). In NS, a few studies have shown the presence of more abundant HDL3 particles than HDL2 particles in the active phase of the disease compared with healthy controls (*9*, *10*). However, these studies did not report data in the remission phase of the disease. In our present study, we demonstrated that paediatric NS patients have different HDL subclass distribution in both the acute and remission phase of the disease. The relative abundance of small HDL particles in the acute phase could be a consequence of the defects in the maturation process of lipid-poor HDL into lipid-rich HDL particles (*9*, *10*). As described recently, abnormalities in HDL composition and function in NS resulted from the deficiency of key molecules involved in HDL metabolism, including lecithin-cholesterol acyltransferase (LCAT), scavenger receptor B1 (SR-B1), hepatic lipase (HL) and increased concentration of cholesteryl ester transfer protein (CETP) and low serum albumin (*2*). On the other hand, the shift towards large-sized HDL subclasses during remission following corticosteroid therapy could be due to the effect of therapy on lipoprotein metabolism (*24*). Pertinent to this, previous studies suggest that corticosteroids increase the activity of lipoprotein lipase (LPL) and reduce the activity of hepatic lipase and CETP, which might explain the observed HDL particles distribution switch towards the large HDL particles profile (*25*). Moreover, Nayak *et al.* found significantly higher serum LCAT activity in children during NS remission than in the acute phase of the disease (*4*). Regarding LDL particles, there are limited data concerning the prevalence of sdLDL in adult nephrotic patients, and to our knowledge, there are no published data in pediatric NS patients (*9*, *26*). Therefore, for the first time we demonstrated a very similar size and subclass distribution of LDL particles in both the acute and in the remission phase of NS with a significant difference in LDL-C concentration. Serum TG is a strong inverse determinant of LDL particle size, which means that higher TG is associated with a higher proportion of smaller LDL particle size (*26*). In agreement with this observation we can speculate that the presence of similar LDL size and subclass distribution during the acute and remission phase could be due to persistent hypertriglyceridemia during remission.

Despite significant improvement in serum lipid profile parameters during NS remission after high dose glucocorticoid therapy, target lipid concentrations were not achieved. Taking into account NHLBI clinical practice guidelines, our study participants in the remission phase had high TC, LDL-C and TG concentrations (*17*). For this reason, a deeper investigation into cholesterol homeostasis pathways in this pathology was necessary. According to our knowledge, we are the first to monitor changes in the concentration of NCS in NS. In our study, 7-DHC/TC and β-sitosterol/TC were significantly increased in the disease remission phase compared with the acute phase, suggesting increased cholesterol synthesis and absorption during remission. A previous study indicated that NCS exhibited specific reciprocal relationships, which means that cholesterol metabolism is regulated by the equivalence between cholesterol absorption and endogenous synthesis (*13*). An inverse relationship between cholesterol intestinal absorption and synthesis is indicative of cholesterol homeostasis in healthy people. Similarly, results of our study show inverse relationship between markers of cholesterol synthesis and absorption, but it did not reach significance. There are limited data about cholesterol synthesis and absorption markers in patients with kidney diseases. The existing data indicate that, in general, higher cholesterol absorption is accompanied by lower cholesterol synthesis in such clinical conditions (*13*). Gylling *et al.* reported that cholesterol absorption in healthy children younger than 10-years-old is a prevalent mechanism compared with cholesterol synthesis in cholesterol homeostasis balance (*14*). We found a significant decrease in desmosterol/β-sitosterol and 7-DHC/β-sitosterol ratios in patients with steroid sensitive NS during disease remission, indicating the positive effects of therapy on improving cholesterol balance. However, the absence of any correlation between cholesterol synthesis and absorption markers suggest that even when patients were in remission, cholesterol homeostasis was not yet achieved.

It is important to emphasize that corticosteroid therapy significantly affects lipoprotein metabolism in NS. Inconsistent results have been reported regarding the effects of corticosteroid treatment on dyslipidaemia (*25*). Our results are in agreement with previous studies (*3*, *4*, *24*, *27*). It is also known that corticosteroids can induce dyslipidaemia by currently unknown mechanisms (*25*). The improved lipid profile in NS patients could be due to corticosteroid effects on podocytes or their anti-inflammatory properties. Several studies have shown that corticosteroid therapy improves the regeneration of podocytes and the restoration of the glomerular filtration barrier which may normalize albumin in serum and ameliorate dyslipidaemia (*28*). This hypothesis is supported by our observation of a significant inverse correlation between serum albumin and cholesterol concentration during remission (data not shown). On the other hand, as NS is an inflammatory disorder and inflammation itself is associated with dyslipidaemia, the anti-inflammatory effect of corticosteroids could result in an improved lipid profile (*29*). However, our study was designed not to compare corticosteroid therapy-related differences in lipid metabolism but rather to determine whether lipid abnormalities were different between the acute and remission phases. A limitation of our study is that the lipid profile was not assessed in the remission phase after glucocorticoid therapy, so our preliminary conclusions need to be confirmed in the paediatric NS patients.

To conclude, in addition to confirming the presence of hyperlipidaemia in the remission phase of NS in children after high-dose glucocorticoid therapy, our data show changes in HDL particle distribution, followed with similar LDL particles size and LDL subclass distribution. Cholesterol metabolism changed towards an increase in cholesterol absorption. The absence of a significant interrelationship between cholesterol synthesis and absorption markers indicated that complete cholesterol homeostasis was not achieved. It is our opinion that remission is followed by favourable changes in the serum lipid profile, HDL particle subclass distribution and cholesterol metabolism in paediatric NS patients. Lipid profile assessment during remission requires additional research.
